# Cognitive and psychosocial outcomes of mechanically ventilated intensive care patients with and without delirium

**DOI:** 10.1186/s13613-020-00723-2

**Published:** 2020-08-03

**Authors:** Daniella Bulic, Michael Bennett, Ekavi N. Georgousopoulou, Yahya Shehabi, Tai Pham, Jeffrey C. L. Looi, Frank M. P. van Haren

**Affiliations:** 1 Faculty of Medicine, University of New South Wales, Sydney, Australia; 2 Prince of Wales Clinical School of Medicine, Faculty of Medicine, University of New South Wales, Sydney, Australia; 3 Australian National University Medical School, Canberra, Australia; 4 Centre for Health and Medical Research, ACT Health Directorate, Canberra, Australia; 5 Monash Health and Monash University, Melbourne, Australia; 6 Interdepartmental Division of Critical Care Medicine, University of Toronto, Toronto, Canada; 7 Keenan Research Center, Li Ka Shing Knowledge Institute, St. Michael’s Hospital, Toronto, Canada; 8 Service de Médecine Intensive-Réanimation, APHP, Hôpital de Bicêtre, Hôpitaux Universitaires Paris-Saclay, Le Kremlin-Bicêtre, France; 9 Academic Unit of Psychiatry and Addiction Medicine, Australian National University Medical School, Canberra, Australia; 10 ICU, Canberra Hospital, Canberra, Australia

**Keywords:** Delirium, Intensive care, Post-traumatic stress disorder, Cognition, Psychosocial function, Long-term outcomes

## Abstract

**Objective:**

Delirium is common in intensive care patients and is associated with short- and long-term adverse outcomes. We investigated the long-term risk of cognitive impairment and post-traumatic stress disorder (PTSD) in intensive care patients with and without delirium.

**Methods:**

This is a prospective cohort study in ICUs in two Australian university-affiliated hospitals. Patients were eligible if they were older than 18 years, mechanically ventilated for more than 24 h and did not meet exclusion criteria. Delirium was assessed using the Confusion Assessment Method for Intensive Care Unit. Variables assessing cognitive function and PTSD symptoms were collected at ICU discharge, after 6 and 12 months: Mini-Mental State Examination, Telephone Interview for Cognitive Status, Impact of Events Scale-Revised and Informant Questionnaire for Cognitive Decline (caregiver).

**Results:**

103 participants were included of which 36% developed delirium in ICU. Patients with delirium were sicker and had longer duration of mechanical ventilation and ICU length of stay. After 12 months, 41/60 (68.3%) evaluable patients were cognitively impaired, with 11.6% representing the presence of symptoms consistent with dementia. When evaluated by the patient’s caregiver, the patient’s cognitive function was found to be severely impaired in a larger proportion of patients (14/60, 23.3%). Delirium was associated with worse cognitive function at ICU discharge, but not with long-term cognitive function. IES-R scores, measuring PTSD symptoms, were significantly higher in patients who had delirium compared to patients without delirium. In regression analysis, delirium was independently associated with cognitive function at ICU discharge and PTSD symptoms at 12 months.

**Conclusions:**

Intensive care survivors have significant rates of long-term cognitive decline and PTSD symptoms. Delirium in ICU was independently associated with short-term but not long-term cognitive function, and with long-term PTSD symptoms.

*Trial registration *Australian New Zealand Clinical Trials Registry, ACTRN12616001116415, 15/8/2016 retrospectively registered, https://www.anzctr.org.au

## Introduction

With improvements in critical care and declining intensive care unit (ICU) mortality, the number of ICU survivors is increasing. These survivors are frequently left with significant long-term complications [1]. Cognitive impairment is an important long-term complication and is associated with both a reduced quality of life and increased healthcare costs and caregiver needs, although the magnitude of the problem is uncertain [2]. In addition, poor mental health and functional disability including depression and post-traumatic stress disorder (PTSD) are common in ICU survivors [3].

Delirium is an acute organic brain dysfunction characterised by disturbances of attention and cognition with a fluctuating course as a direct consequence of an underlying medical condition [4]. It occurs in different healthcare settings [5], affecting between 15 and 20% of general hospital patients, and up to 80% of patients in an ICU [6, 7]. Delirium has been associated with long-term disability following non-ICU hospitalisations [8–10] and with poor outcomes following ICU admission including prolonged length of stay, cognitive impairment after hospital discharge [11–14], and increased odds of long-term disability in activities of daily living [15]. Although suggested otherwise in the past, delirium is not associated with short-term mortality in critically ill patients, except for an increase in 90-day mortality associated with the mixed delirium subtype [16–18]. Defining the extent of the association between delirium and persistent cognitive impairments in critically ill patients has been identified as an important research priority due to the high prevalence of both conditions [19].

We hypothesised that the occurrence of delirium in ICU is associated with long-term effects on cognition and psychosocial function and with symptoms of PTSD [20].

## Methods

### Design and setting

This is a multicentre prospective cohort study between October 2012 and June 2016 in mixed ICUs of two large university-affiliated hospitals in Australia: the Canberra Hospital in the Australian Capital Territory and the Prince of Wales Hospital in Sydney, New South Wales. The full study protocol has been previously published [21].

The study was prospectively approved by the institutional Ethics Committees [ACT Health Human Research Ethics Committee (ETH.6.12.130), and South Eastern Health Human Research Committee (HREC/I2/242POWH/460)] and was retrospectively registered with the Australian New Zealand Clinical Trial Registry (ACTRN12616001116415).

### Population

Patients were eligible for the study if they were 18 years or older and received mechanical ventilation for more than 24 h. Patients were excluded if one of the following was present: admission with a neurological diagnosis, e.g. stroke, neurotrauma; end stage or acute liver failure; culturally and linguistically diverse background with insufficient literacy in the English language; death was deemed imminent and inevitable; patient was a nursing home resident; physical and/or cognitive decline before the ICU admission was reported by the patient, family or documented in the patient’s medical record. Patients were also excluded if they died during their ICU admission.

Written informed consent was obtained prior to enrolment. For patients who were unable to give informed consent because of their health status (mechanical ventilation and sedation), a substitute decision maker or caregiver was approached. The caregiver was selected by interviewing the individuals nominated as the next of kin and establishing the most appropriate person to take this role. This consent included their later involvement in the assessment of patients’ overall function at the 12-month follow-up. When patients previously unable to consent became capable of doing so, consent was obtained.

Consecutive patients were enrolled in the study only on days that study personnel were available to obtain written consent.

### Exposure

Patients received no intervention other than standard ICU care during their stay. The Richmond Agitation and Sedation Score (RASS) was administered four-hourly to assess sedation [22, 23] and the Confusion Assessment Method for Intensive Care Unit (CAM-ICU) was performed twice a day [24]. Patients with a RASS between –2 and +3 were administered the CAM-ICU to test for the presence of delirium [25]. The CAM-ICU was performed by trained bedside intensive care nurses in one hospital as a routine element of patient care and monitoring, and by a dedicated trained intensive care doctor involved with the study in the other hospital. The 2014 updated version of the CAM-ICU is valid according to DSM-5 criteria and reliable regarding inter-observer agreement in a research setting [26]. Delirium is determined by the presence or absence of four features: (a) acute change or a fluctuation in mental status; (b) inattention; (c) disorganised thinking; and (d) altered level of consciousness.

Based on the CAM-ICU results, patients were divided into two groups: the CAM-ICU positive group that tested positive at any time whilst mechanically ventilated; and the CAM-ICU negative group of patients who never tested delirium positive during their mechanical ventilation. Of note, both hospitals did not have delirium prevention bundles in place during the time the study was conducted.

### Data collection

Patients completed specific tests administered by a mental health social worker on three occasions. Permission to use these tests was obtained through direct contact with authors. The tests, which can be found in Additional file 1: Appendix 1–4, consisted of the Mini-Mental State Examination (MMSE) at discharge from ICU; and the Impact of Events Scale-Revised (IES-R), and the Telephone Interview for Cognitive Status (TICS) at 6 and 12 months after discharge. The caregiver completed the Informant Questionnaire for Cognitive Decline (IQCODE) at 12 months after the patient’s discharge. All tests were administered by telephonic consultation, except the MMSE at ICU discharge, which was performed face-to-face. The tests employed are established and validated in assessing cognition and psychosocial function. In utilising the caregiver, we gained an independent insight into patients’ psychosocial abilities at 12 months after discharge from ICU as compared with their pre-ICU psychosocial abilities.

The MMSE is commonly used in measuring cognitive function in hospitalised patients [27], and is a scale with a score from 0 to 30. Lower scores indicate poorer performance (< 10 = severe impairment, 10–20 = moderate impairment, 21–24 = mild impairment, 25–30 = normal). The accepted cut-off to warrant further investigation is a score of  < 25, and represents the presence of symptoms consistent with dementia [28].

The TICS is a modified telephone version of MMSE, an 11-item screening test (maximum score 41 points) developed for the assessment of cognitive function of patients who are unable to be assessed in person [29]. Lower scores indicate poorer performance (< 20 = severe impairment; 20–25 = mild impairment; 26–32 = ambiguous; > 32 = normal). The cut-off for the TICS is < 26, which represents the presence of symptoms consistent with dementia [30].

The IES-R is a measure designed to assess subjective distress for any specific life event. [31]. It consists of three subscales: hyperarousal, intrusion and avoidance, which parallel the DSM-5 criteria for PTSD [4, 32]. IES-R has 22 questions with the responses scored on a scale from 0 to 4. Higher scores indicate more distress (< 23 = not distressed; 23–37 = probable diagnosis of PTSD; > 37 = severe PTSD). Although the IES-R was originally not intended to be used for screening and/or the assessment of a diagnosis of PTSD, it is currently one of the most widely used measures to assess post-traumatic stress symptoms [33]. The IES-R has been shown to be an excellent brief PTSD symptom measure and screening tool in intensive care survivors when compared with the Clinician-Administered PTSD Scale (CAPS), which is the current state-of-the-art PTSD diagnostic reference standard [34.]

The IQCODE is designed to assess cognitive impairment in older people [35, 36]. The test is widely used in conjunction with other cognitive assessments, and with no age limitations. The short IQCODE consists of 16 questions with the responses scored on a scale from 1 = much improved; 2 = a bit improved; 3 = not much change; 4 = a bit worse; 5 = much worse [range 16 (not impaired) to 80 (severely impaired)]. Patients with a score of ≥ 54 are considered to have symptoms consistent with dementia [37.]

### Statistical analysis

All statistical analysis was done using Statistical Package for the Social Sciences (SPSS) Research Engine, Version 24.0 IBM SPSS Statistics (2017), and “R” version 3.3.3. We calculated our sample size based on expected outcomes for MMSE. We calculated that, at a type-I error rate of 5% (alpha 0.05), we could find the clinically significant difference of two points on the MMSE [8] between groups with 80% power if we included 81 patients in each group. Continuous variables were reported as mean ± standard deviation (SD) or median [1st, 3rd quartiles] and categorical variables as count and proportion. Normality of the data distribution was visually assessed by means of histograms. Comparisons of proportions were made using the Pearson’s Chi square or the Fisher’s exact test. Continuous variables were compared between delirium status groups using Students’ *t* test or the Mann–Whitney U test depending on the distribution of that data. Multivariable linear regression was undertaken to investigate the independent predictors of cognitive function and PTSD, using the following potential predictors: delirium, age, sex, APACHE II (in a separate model where age and sex were removed due to collinearity issues). To address that statistical power was not met, we performed bootstrapping for 1000 samples with Bias corrected accelerated 95% Confidence Intervals, to check the stability of the results. For all analyses, level of statistical significance was set at alpha = 0.05.

## Results

**Fig. 1:**
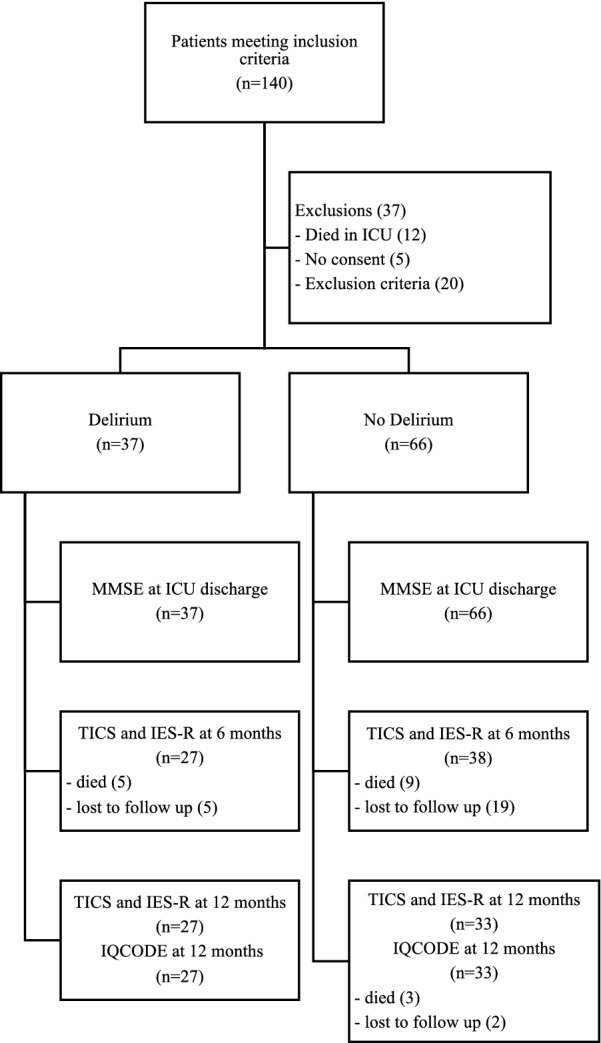
Study flow chart

A total of 103 patients were enrolled. The trial was stopped because of low recruitment rates. The main reasons for recruitment failure were staffing issues and unresolvable organisational problems at one recruitment site. The study flowchart is shown in Fig. 1. There were 26 patients lost to follow up, with 21 patients who were not contactable by telephone despite a minimum of 3 attempts, and 5 patients who withdrew from the study.

### Demographic and clinical characteristics

The demographic and clinical characteristics of patients with and without delirium are summarised in Table 1. Delirium affected 37 patients (36%) during their period of mechanical ventilation in ICU. Patients with delirium were sicker compared to patients without delirium (APACHE II 23 ± 8 vs 18 ± 7, *p* = .002), spent more hours on mechanical ventilation (144 [72–258] vs 62 [40–119], *p* < .001), had a longer ICU length of stay (199 h [165–479] vs 150 [101–265], *p* = .001) but similar 1-year mortality.

**Table 1:** Demographic and clinical characteristics of all patients, and of patients with and without delirium during ICU stay

Variable	All patients (*n* = 103)	No delirium (*n* = 66)	Delirium (*n* = 37)	*P* value*
Age (years), mean (SD)	60 (16)	60 (17)	62 (14)	0.52
Gender (male), *n* (%)	53 (52%)	36 (55%)	17 (46%)	0.40
APACHE II, mean (SD)	20 (7)	18 (7)	23 (8)	*0.002*
Admission (acute), *n* (%)	90 (87%)	59 (89%)	31 (84%)	0.18
Ventilation hours, median (IQR)	82 (44–167)	62 (40–119)	144 (72–258)	*< 0.001*
ICU LOS hours, median (IQR)	173 (116–304)	150 (101–265)	199 (165–479)	*0.001*
1-year mortality, *n* (%)	17 (17%)	12 (18%)	5 (14%)	0.59

Italic values are statistically significant

*APACHE* Acute physiology age and chronic health evaluation score, *ICU LOS* Intensive care length of stay

*P values between patients with and with no delirium derived from Independent Samples *t* test or Mann–Whitney *U* test

### Cognitive and psychosocial outcomes

**Fig. 2:**
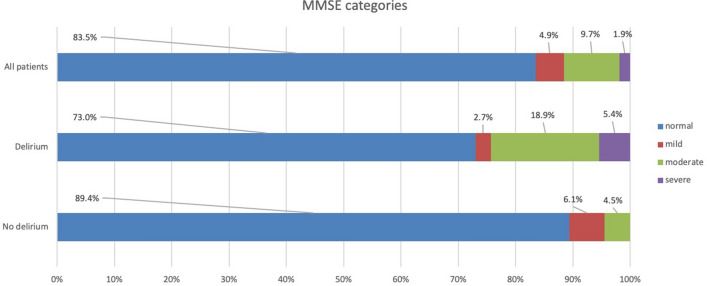
Frequency distribution of MMSE categories at ICU discharge in all patients, and in patients with and without delirium during ICU stay

Variables related to cognitive and psychosocial outcomes are shown in Table 2. At ICU discharge, there were 17 patients (16.5%) with an MMSE score of  < 25, indicating cognitive impairment. There was no difference in median MMSE scores between patients with and without delirium (Table 2), but patients with delirium were more often in the moderate or severe category (*p* = 0.015, Fig. 2). In multivariable linear regression analysis examining all patients, age and delirium were independently associated with lower MMSE at ICU discharge (*b* = −0.076 95%CI −0.129, −0.022; *p* = 0.006 and *b* = −2.38 95%CI −4.09, −0.672; *p* = 0.007, respectively, after adjusting for sex; Additional file 1: Appendix 5).

**Table 2:** Cognitive and psychosocial outcomes of all patients, and of patients with and without delirium during ICU stay

Variable at ICU discharge	All patients (*n* = 103)	No delirium (*n* = 66)	Delirium (*n* = 37)	*P* value*
MMSE, median (IQR)	28 (26–30)	29 (27–30)	27 (24–29)	0.17

Variables after 6 months	(*n* = 65)	(*n* = 38)	(*n* = 27)	
TICS, median (IQR)	32 (30–34)	32 (30–35)	32 (30–33)	0.97
IES-R, median (IQR)	20 (6–34)	20 (3–33)	21 (8–36)	0.50

Variables after 12 months	(*n* = 60)	(*n* = 33)	(*n* = 27)	
TICS, median (IQR)	31 (28–34)	30 (28–34)	31 (29–34)	0.76
IES-R, median (IQR)	15 (4–39)	11 (2–27)	30 (10–51)	*0.028*
IQCODE, median (IQR)	50 (48–53)	50 (48–53)	51 (48–55)	0.78

Italic value is statistically significant

*MMSE* Mini-mental state examination, *TICS* Telephone interview for cognitive status, *IES-R* impact of events scale-revised, *PTSD* Post-traumatic stress disorder, *IQCODE* Informant questionnaire for cognitive decline in the elderly

*P values between patients with and with no delirium derived from Mann–Whitney *U* test

**Fig. 3:**
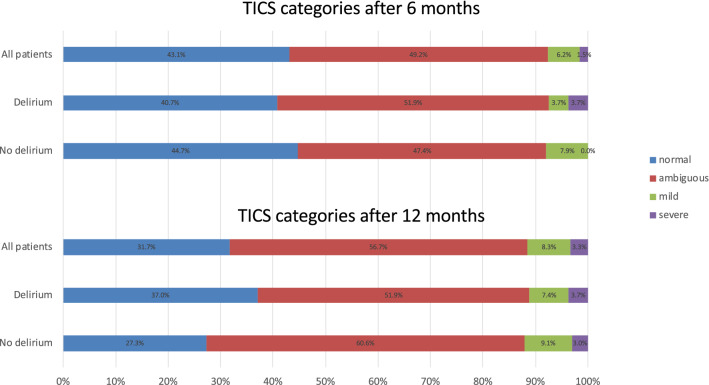
Frequency distribution of TICS categories after 6 and 12 months in all patients, and in patients with and without delirium during ICU stay

At 6 months, the majority of evaluable patients (37/65, 56.9%) had evidence of cognitive impairment evidenced by an abnormal TICS score, with 7.7% representing the presence of symptoms consistent with dementia (Fig. 3). At 12 months, 41/60 (68.3%) had an abnormal TICS score, with 11.6% representing the presence of symptoms consistent with dementia (Fig. 3). There were no differences between patients with and without delirium in ICU regarding TICS scores or categories after 6 and 12 months. When evaluated by the caregiver at 12 months (IQCODE), cognitive function was severely impaired (representing the presence of symptoms consistent with dementia) in a higher proportion of patients (14/60, 23.3%), with no difference between patients with or without delirium in ICU (29.6% vs 18.2%, *p* = 0.230).

**Fig. 4:**
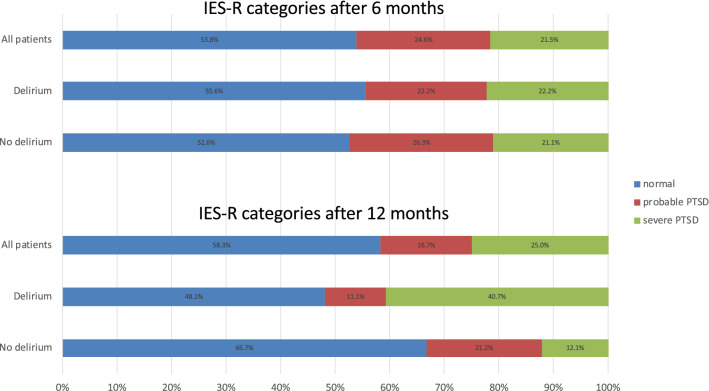
Frequency distribution of PTSD severity (IES-R) after 6 and 12 months in all patients, and in patients with and without delirium during ICU stay

IES-R scores indicating PTSD symptoms were present in 30/65 (46.1%) and 25/60 (41.7%) of evaluable patients after 6 and 12 months, respectively. The frequency distribution of PTSD severity is shown in Fig. 4. After 6 months, there was no statistically significant difference in IES-R or PTSD category distribution between patients with and without delirium. After 12 months, patients who had delirium in ICU had significantly higher IES-R scores than patients without (*p* = 0.028, Table 2). On multivariable linear regression analysis, delirium in ICU was independently associated with IES-R at 12 months; patients with delirium had 2 units higher score in IES-R, *p* = 0.047 (Table 3). Disease severity as measured by APACHE II was not independently associated with IES-R scores after 12 months (Additional file 1: Appendix 6).

**Table 3:** Multivariable linear regression models exploring the role of delirium status on PTSD symptoms (IES-R) after 6 and 12 months

	IES-R 6 months				IES-R 12 months			
	(n = 65)		(Bootstrapping 1000 samples)		(n = 60)		(Bootstrapping 1000 samples)	
Variable	*b* (95%CI)	*P* value	*b* (95%CI)	*P* value	*b* (95%CI)	*P* value	*b* (95%CI)	*P* value
Univariable model								
Delirium (yes vs. no)	0.096 (−0.472, 0.663)	0.737	0.096 (−0.460, 0.592)	0.738	0.691 (0.102, 1.28)	0.022	*0.691 (0.136, 1.23)*	*0.027*
Multivariable model								
Age (per 1 year)	−0.007 (−0.028, 0.014)	0.516	−0.007 (−0.031, 0.015)	0.499	−0.018 (−0.042, 0.006)	0.144	−0.018 (−0.040, 0.003)	0.157
Gender (male vs. female)	−0.353 (−0.935,0.229)	0.229	−0.353 (−0.895,0.194)	0.206	−0.368 (−0.957,0.221)	0.215	−0.368 (−0.959, 0.243)	0.204
Delirium (yes vs. no)	0.038 (−0.536, 0.611)	0.896	0.038 (−0.446, 0.483)	0.880	*0.578 (−0.013, 1.17)*	*0.055*	*0.578 (0.036, 1.06)*	*0.047*

Dependent variables have been Ln-transformed due to lack of normality. Coefficients presented in the Table refer to the association between the independent variables and the Ln-transformed dependent variables

Italic values are statistically significant

## Discussion

In this prospective long-term follow-up study of intensive care survivors, we found an association between the occurrence of delirium during ICU admission and cognitive impairment at ICU discharge, but not after 6 or 12 months. Cognitive impairment was prevalent and severe in a significant proportion of intensive care survivors, with 1 in 6 patients at ICU discharge and 1 in 9 patients after 12 months exhibiting symptoms of severe cognitive impairment consistent with dementia. We found the prevalence of cognitive impairment improved at 12 months compared to at ICU discharge, similar to a previous report [38]. However, this observation may be confounded by the possibility that early cognitive assessment reflects residual pain, the effects of analgesic and sedative drugs, and/or residual delirium [38, 39].

Interestingly, according to scores obtained from the caregiver, a much larger proportion—almost 1 in 4 patients—showed severe cognitive impairment after 12 months. The observation that patients assessed their cognition differently to their caregiver, usually rating themselves higher, suggests they may have had an unrealistic view of their cognitive state. This phenomenon, called ‘anosognosia’ (the unawareness of deficits), is common in cognitive impairment and dementia. Alternatively, their caregiver may have underestimated the patient’s true cognitive ability. Regardless, this observation underlines the importance of interviewing patients’ caregivers when assessing long-term cognitive outcomes. This observation is consistent with another study which showed that patients assessed their cognition differently to the ICU team whilst in the ICU and highlights that the link between cognition and ICU memories may not be not reliable [26].

In addition, we found post-traumatic stress symptoms were common after 6 and 12 months, and often severe. The occurrence of delirium during ICU admission was associated with PTSD symptoms after 12 months, independent of age, sex, and disease severity,

Our findings are different from those of a previous study, which did not show an association between ICU delirium and PTSD symptoms [40]. There are several important methodological differences that could provide possible explanations for this discrepancy. First, the studies used different tools for the detection of PTSD symptoms. Second, we compared the distribution of IES-R scores between the groups rather than simply assigning groups based on a fixed cut-off (PTSD present or absent). In our opinion, because the IES-R is a continuous variable, a shift in the distribution of scores is the most relevant outcome because higher scores are consistent with more symptoms, regardless of which cut-off is used to define PTSD. Third, the study by Svenningsen et al. used a shorter duration of follow-up (6 months). In our cohort, there was no statistically significant difference in PTSD symptom scores after 6 months. However, after 12 months the patients who had ICU delirium had higher IES-R scores than patients who never had ICU delirium. Finally, there was a difference in the use of physical restraints between the studies. Although we did not collect data on this variable, in both hospitals where our study was performed, the use of physical restraints for agitated patients is reportedly common and standard practice; physical restraints were not used in the study by Svenningsen et al. The use of physical restraints has been associated with a higher incidence of delirium, development of PTSD and recall of delusions [41, 42].

PTSD is likely a serious problem in survivors of critical illness, and could significantly impact on long-term health-related quality of life [43, 44]. Our study suggests an association between delirium during ICU admission and later development of symptoms of PTSD, but this observation needs to be interpreted with caution. The screening test employed does not represent an expert clinical diagnosis of PTSD (which requires a qualified mental health professional diagnostic assessment), but rather an indication that the patients met criteria for such a diagnosis based on the existing literature for the IES-R test. We dichotomised patients in groups based on whether they had an episode of delirium in ICU but did not assess the duration or burden of ICU delirium. The latter has been independently associated with functional long-term outcomes [15, 45].

Regardless, it is conceivable that ICU delirium as a marker of organic brain dysfunction may be a risk factor for the development of PTSD. For example, the development of acute PTSD-related symptoms in the first months after ICU admission appears to be related to recall and memories of delusions, whereas memories of real events during critical illness may give some protection from development of anxiety [46]. Other studies also suggest memories of frightening and/or psychotic experiences consistently predicted post-ICU PTSD [43]. Detection, prevention and early treatment of potentially modifiable factors, e.g. by offering psychological support in ICU or by providing patients with ICU diaries, may have the potential to prevent PTSD and its associated burden [47, 48].

Both the development of ICU delirium and post-ICU PTSD have been linked to the administration of benzodiazepines in ICU [43, 49, 50]. This may provide a potential link between the occurrence of these disturbances, although causality would be difficult to prove. We did not assess the type and dose of sedation used in our cohort. Importantly, the severity of critical illness was not associated with the development of long-term PTSD in our study, which is consistent with the literature [43]. We also did not find an association between age, delirium and PTSD symptoms. It has been suggested that whilst older age is an independent risk factor for the development of delirium in ICU [51–53], older age may also provide a degree of protection against the development of PTSD in medically ill patients [54].

The strengths of our study include the employment of a set of standardised validated assessments of patients’ cognitive and psychosocial function, and the extended follow-up after ICU discharge, which enabled us to complete the long-term assessment of cognitive and psychosocial outcomes necessary to explore PTSD symptomatology [4, 55] and to allow PTSD symptoms to surface. In addition, by incorporating cognitive evaluation by the patients’ caregivers, we were able to obtain a more complete picture of the long-term cognitive function in these intensive care survivors.

Our study also has several limitations. First, as previously indicated, we were unable to enrol the sample needed to have sufficient power to detect expected differences in long-term cognitive function between patients with and without delirium. In addition, there was a poor retention rate at 12 months because of death and lost to follow up, further decreasing the power of our analysis. Consequently, the results of our study should be viewed as hypothesis-generating. Second, we were unable to test patients’ cognition before ICU admission and critical illness. We addressed baseline cognition before ICU admission by excluding patients whose cognition was known to be compromised before their admission, as well as patients from CALD background who were likely to, in times of stress, revert to their native language [56, 57]. We were also unable to test for symptoms of depression or anxiety before ICU admission. Third, we may have underestimated the prevalence of cognitive impairment because we did not use extensive neuropsychological testing [2]. Fourth, as mentioned earlier, we did not assess the duration (or burden) of ICU delirium, which has been independently associated with functional long-term outcomes. We also did not collect data on the cumulative doses of sedatives and opiates, which have been shown to have a potential impact of delirium recognition [25]. Finally, as with any observational study, we were not able to address the possibility of confounding by death or withdrawal, and/or exclude the possibility of bias due to unmeasured confounders.

In conclusion, in a cohort of mechanically ventilated ICU patients, both cognitive impairment and PTSD symptoms were common. Delirium during ICU admission was independently associated with short-term but not long-term cognitive function, and with long-term PTSD symptoms. Further studies are needed to determine if delirium directly affects the trajectory of development of PTSD and to identify potentially treatable and modifiable factors.

## Supplementary information

**Additional file 1: Appendix 1.** Mini-mental state examination**. Appendix 2.** Telephone interview for cognitive status. **Appendix 3**. Impact of events scale-revised**. Appendix 4.** Informant questionnaire for cognitive decline in the elderly**. Appendix 5.** Results regression analysis of factors associated with MMSE outcome**. Appendix 6.** Results regression analysis of factors (delirium status, APACHE II) with PTSD (IES-R) outcome after 12 months.

## Data Availability

The dataset analysed during the current study is available from the corresponding author on reasonable request.

## Abbreviations

APACHE II: Acute physiological and chronic health evaluation II
ACTRN: Australian New Zealand Clinical Trial Registration Number
CALD: Culturally and linguistically diverse
CAM-ICU: Confusion assessment method for intensive care unit
CAPA: Cognitive and psychological assessment
DSM: Diagnostic statistical manual
HREC: Human research committee
ICU: Intensive care unit
IES-R: Impact of events scale-revised
IQCODE: Informant questionnaire on cognitive decline
IQR: Interquartile range
MMSE: Mini-mental state examination
PTSD: Post-traumatic stress disorder
RASS: Richmond agitation scale score
SPSS: Statistical package for the social sciences
TICS: Telephone interview for cognitive status
