# Supplementary material for: Cognitive and psychosocial outcomes of mechanically ventilated intensive care patients with and without delirium
Source: Ann Intensive Care. 2020 Aug 3;10:104. doi: 10.1186/s13613-020-00723-2 (PMC7399009; doi:10.1186/s13613-020-00723-2)
Supplement: Supplementary file 1 — Additional file 1: Appendix 1. Mini-mental state examination. Appendix 2. Telephone interview for cognitive status. Appendix 3. Impact of events scale-revised. Appendix 4. Informant questionnaire for cognitive decline in the elderly. Appendix 5. Results regression analysis of factors associated with MMSE outcome. Appendix 6. Results regression analysis of factors (delirium status, APACHE II) with PTSD (IES-R) outcome after 12 months. [file 13613_2020_723_MOESM1_ESM.pdf]

Mini-Mental State Examination (MMSE)

Patient's Name: ____ Date: _____________

Instructions: Score one point for each correct response within each question or activity.

Maximum Score	Patient's Score	Questions
5		"What is the year? Season? Date? Day? Month?"
5		"Where are we now? State? County? Town/city? Hospital? Floor?"
3		The examiner names three unrelated objects clearly and slowly, then the instructor asks the patient to name all three of them. The patient's response is used for scoring. The examiner repeats them until patient learns all of them, if possible.
5		"I would like you to count backward from 100 by sevens." (93, 86, 79, 72, 65, …)<br>Alternative: "Spell WORLD backwards." (D-L-R-O-W)
3		"Earlier I told you the names of three things. Can you tell me what those were?"
2		Show the patient two simple objects, such as a wristwatch and a pencil, and ask the patient to name them.
1		"Repeat the phrase: 'No ifs, ands, or buts.'"
3		"Take the paper in your right hand, fold it in half, and put it on the floor." (The examiner gives the patient a piece of blank paper.)
1		"Please read this and do what it says." (Written instruction is "Close your eyes.")
1		"Make up and write a sentence about anything." (This sentence must contain a noun and a verb.)
1		"Please copy this picture." (The examiner gives the patient a blank piece of paper and asks him/her to draw the symbol below. All 10 angles must be present and two must intersect.)<br>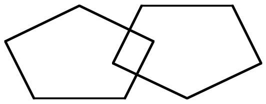
30		TOTAL

Interpretation of the MMSE:

**Method**	**Score**	**Interpretation**
Single Cutoff	<24	Abnormal
Range	<21	Increased odds of dementia
	>25	Decreased odds of dementia
Education	21	Abnormal for 8th grade education
	<23	Abnormal for high school education
	<24	Abnormal for college education
Severity	24-30	No cognitive impairment
	18-23	Mild cognitive impairment
	0-17	Severe cognitive impairment

Interpretation of MMSE Scores:

**Score**	**Degree of Impairment**	**Formal Psychometric Assessment**	**Day-to-Day Functioning**
25-30	Questionably significant	If clinical signs of cognitive impairment are present, formal assessment of cognition may be valuable.	May have clinically significant but mild deficits. Likely to affect only most demanding activities of daily living.
20-25	Mild	Formal assessment may be helpful to better determine pattern and extent of deficits.	Significant effect. May require some supervision, support and assistance.
10-20	Moderate	Formal assessment may be helpful if there are specific clinical indications.	Clear impairment. May require 24-hour supervision.
0-10	Severe	Patient not likely to be testable.	Marked impairment. Likely to require 24-hour supervision and assistance with ADL.

Source:

- Folstein MF, Folstein SE, McHugh PR: "Mini-mental state: A practical method for grading the cognitive state of patients for the clinician." *J Psychiatr Res* 1975;12:189-198.

Appendix 2. This appendix was part of the submitted manuscript and has been peer reviewed. It is posted as supplied by the authors

APPENDIX 2

TICS™ Telephone Interview for Cognitive Status™

Record Form

by Jason Brandt, PhD, and Marshal F. Folstein, MD

Examinee Name or ID ______________________ Date of Testing ___/___/___ Date of Birth ___/___/___ Age ______

Address of Examinee ____
Street City

____ Education Level (no. of years or degree) ________________
State Zip Code

Name of Examiner ______________________________ Name of Proctor ______________________

Suggested Qualitative Interpretive Ranges for TICS Total Score Range

- ☐ 33-41 Nonimpaired
- ☐ 26-32 Ambiguous
- ☐ 21-25 Mildly Impaired
- ☐ ≤ 20 Moderately to Severely Impaired

TICS *T* scores

For individuals with < 12 years of education and ≥ 65 years of age	For individuals with ≥ 12 years of education and ≥ 60 years of age
MMSE Total score equivalent of TICS Total score = _________ (Obtain from Table 3 of TICS Professional Manual)<br><br>*T* score = _________ (Obtain from Tables A10-A14 of MMSE Clinical Guide)	*T* score = _________ (Obtain from Table 2 of TICS Professional Manual)<br><br>90% Confidence Interval _________ to _________ (*T* score – 8) (*T* score + 8)

PAR • 16204 N. Florida Ave. • Lutz, FL 33549 • 1.800.331.8378 • www.parinc.com

Copyright © 1987, 2003 by PAR. All rights reserved. May not be reproduced in whole or in part in any form or by any means without written permission of PAR. This form is printed in blue and black ink on white paper. Any other version is unauthorized.

9 8 7 6 5 Reorder #RO-5182 Printed in the U.S.A.

WARNING! PHOTOCOPYING OR DUPLICATION OF THIS FORM WITHOUT PERMISSION IS A VIOLATION OF COPYRIGHT LAWS.

TICS Administration Instructions

Prior to the TICS administration, examiners should ensure that they have obtained all of the necessary information (e.g., examinee's full name, complete address, etc.).

Instructions to Proctor

In a couple of minutes, I am going to be asking [*examinee's name*] a number of different questions to test [*his/her*] thinking and memory. Before we start, I need to ask you whether the address I have for your current location is correct. Please don't repeat it out loud if [*examinee's name*] is in the room with you, since I will be asking [*him/her*] the same question in a few minutes. Is your current address [*examinee's address*]? If the answer is "no," please ask the proctor either to step into another room before giving you the correct address or to have the examinee leave the room briefly before giving you the address. Then go on to say, **Please be sure that all papers, pencils, books, calendars, newspapers, and everything else that might provide distraction or visual cues are removed from [*examinee's name*] sight. Also, please be sure that the room is quiet; there should be no television, radio, or music playing.**

Some of the questions may be difficult for [*examinee's name*] to answer. [*He/She*] may ask you for help. If [*he/she*] does, just encourage [*him/her*] to do as well as [*he/she*] can. [*He/She*] should guess if necessary. Please do not give [*him/her*] any answers or hints. O.K.? If you and [*examinee's name*] are ready, please put [*him/her*] on the phone.

Instructions to Examinee

I am going to ask you some questions to test your memory. Some of these are likely to be easy for you, but some may be difficult. Please bear with me and try to answer all the questions as best you can. If you can't answer a question, don't worry. Just try your best. Are you ready? These instructions may be repeated *verbatim* or paraphrased, if necessary. For each of the actual TICS items, except Item 5 and Item 8, single repetitions are permitted.

Item	Item response	Scoring criteria	Max. score	Item score
1. **Please tell me your full name.**		1 point for correct first name (or nickname) and 1 point for correct last name	2	
2. **What is today's date?** Probe for month, date, year, day of week, and season if any not provided spontaneously (e.g., **What day of the week is it?** *or* **What season is it?**).		1 point each for precisely correct month, date, year, day of the week, and season (e.g., a hot day in early June is *not* summer)	5	
3. **Where are you right now?** Probe for house number, street, city, state, and zip code if any not provided spontaneously (e.g., **What number is that? What is your zip code?**).		1 point each for correct house number, street, city, state, and zip code (5-digit zip code is sufficient). If examinee is in a facility with no house number (e.g., hospital, nursing home), the name of the facility may be substituted for the house number.	5	
4. **Please count backward from 20 to 1.** If examinee makes an error, ask him or her to try again.		2 points if completely correct on first trial<br>1 point if completely correct on second trial	2	
5. **I am going to read you a list of 10 words. Please listen carefully and try to remember them. When I am done, tell me as many of the words as you can, in any order. Ready? The words are** (*pause*) **cabin, pipe, elephant, chest, silk, theater, watch, whip, pillow, giant.** (*Pause.*) **Now tell me all the words you can remember.** The words should be read at approximately one word every 2 seconds. <u>No repetitions of the word list are permitted.</u>		1 point for each correctly recalled word<br>0 points for incorrect responses, repetitions, or intrusions	10	

Item	Item response	Scoring criteria	Max. score	Item score
6. **I would like you to take the number 100 and subtract 7.** (Pause for a response.) **Now keep subtracting 7 from the answer until I tell you to stop.** No further prompts or instructions are given, except to **"keep going."** Stop the examinee after five serial subtractions.		1 point for each correct subtraction<br>Do not inform examinee of incorrect responses, but allow subtractions to be made from the last response.<br>For example, "93, 85, 78, 71, 65" would be awarded 3 points.	5	
7. **What do people usually use to cut paper?** (Pause for a response.)<br>**How many things are in a dozen?** (Pause for a response.)<br>**What do you call the prickly green plant that lives in the desert?** (Pause for a response.)<br>**What animal does wool come from?**		1 point each for "scissors" or "shears"<br>1 point for "12"<br>1 point for "cactus"<br>1 point for "sheep" or "lamb"	4	
8. **Please repeat this after me: "No ifs, ands, or buts."** (Pause for a response.)<br>**Now, please repeat this after me: "Methodist Episcopal."**<br>No repetitions of the phrases are permitted.		1 point for correct repetition<br>1 point for correct repetition	2	
9. **Who is the President of the United States right now?** (Pause for a response.)<br>**Who is the Vice-President?**<br>Both first and last names must be correct. If only the last name is given, probe for the full name.		1 point for current president's full name<br>1 point for current vice-president's full name	2	
10. **With your finger, tap five times on the part of the phone you speak into.**<br>If the TICS is being administered in person, the examinee should be asked to tap on the table rather than on a telephone receiver.		2 points if five taps are clearly heard<br>1 point if either more than or fewer than 5 taps are heard<br>0 points if no taps are heard.	2	
11. **I am going to say a word and I want you to give me its opposite. For example, if I said "hot," you would say "cold."**<br>**What is the opposite of "west"?** (Pause for a response.)<br>**What is the opposite of "generous"?**		1 point for "east"<br>1 point for "cheap," "stingy," "tight," "selfish," "greedy," "mean," "meager," or other correct antonym	2	

TICS Total score
Total possible points = 41

IMPACT OF EVENTS SCALE-Revised (IES-R)

INSTRUCTIONS: Below is a list of difficulties people sometimes have after stressful life events. Please read each item, and then indicate how distressing each difficulty has been for you DURING THE PAST SEVEN DAYS with respect to ____(event) that occurred on ______________________(date). How much have you been distressed or bothered by these difficulties?

	Not at all	A little bit	Moderately	Quite a bit	Extremely
1. Any reminder brought back feelings about it	0	1	2	3	4
2. I had trouble staying asleep	0	1	2	3	4
3. Other things kept making me think about it.	0	1	2	3	4
4. I felt irritable and angry	0	1	2	3	4
5. I avoided letting myself get upset when I thought about it or was reminded of it	0	1	2	3	4
6. I thought about it when I didn't mean to	0	1	2	3	4
7. I felt as if it hadn't happened or wasn't real.	0	1	2	3	4
8. I stayed away from reminders of it.	0	1	2	3	4
9. Pictures about it popped into my mind.	0	1	2	3	4
10. I was jumpy and easily startled.	0	1	2	3	4
11. I tried not to think about it.	0	1	2	3	4
12. I was aware that I still had a lot of feelings about it, but I didn't deal with them.	0	1	2	3	4
13. My feelings about it were kind of numb.	0	1	2	3	4
14. I found myself acting or feeling like I was back at that time.	0	1	2	3	4
15. I had trouble falling asleep.	0	1	2	3	4
16. I had waves of strong feelings about it.	0	1	2	3	4
17. I tried to remove it from my memory.	0	1	2	3	4
18. I had trouble concentrating.	0	1	2	3	4
19. Reminders of it caused me to have physical reactions, such as sweating, trouble breathing, nausea, or a pounding heart.	0	1	2	3	4
20. I had dreams about it.	0	1	2	3	4
21. I felt watchful and on-guard.	0	1	2	3	4
22. I tried not to talk about it.	0	1	2	3	4

Total IES-R Score:________________

INT: 1, 2, 3, 6, 9, 14, 16, 20
AVD: 5, 7, 8, 11, 12, 13, 17, 22
HYP: 4, 10, 15, 18, 19, 21

Weiss, D.S. (2007). The Impact of Event Scale-Revised. In J.P. Wilson, & T.M. Keane (Eds.) *Assessing psychological trauma and PTSD: a practitioner's handbook* (2nd ed., pp. 168-189). New York: Guilford Press.

Revised Impact of Event Scale (22 questions):

The revised version of the Impact of Event Scale (IES-r) has seven additional questions and a scoring range of 0 to 88.

On this test, scores that exceed 24 can be quite meaningful. High scores have the following associations.

Score (IES-r) **Consequence**

24 or more	PTSD is a clinical concern.[6] Those with scores this high who do not have full PTSD will have partial PTSD or at least some of the symptoms.
33 and above	This represents the best cutoff for a probable diagnosis of PTSD.[7]
37 or more	**This is high enough to suppress your immune system's functioning** (even 10 years after an impact event).[8]

The IES-R is very helpful in measuring the affect of routine life stress, everyday traumas and acute stress

References:

1. Horowitz, M. Wilner, N. & Alvarez, W. (1979). Impact of Event Scale: A measure of subjective stress. Psychosomatic Medicine, 41, 209-218.
2. Weiss, D.S., & Marmar, C.R. (1997). The Impact of Event Scale-Revised. In J.P. Wilson & T.M. Keane (Eds.), Assessing Psychological Trauma and PTSD (pp.399-411). New York: Guilford.
3. Hutchins, E. & Devilly, G.J. (2005). Impact of Events Scale. Victim's Web Site. http://www.swin.edu.au/victims/resources/assessment/ptsd/ies.html
4. Coffey, S.F. & Berglind, G. (2006). Screening for PTSD in motor vehicle accident survivors using PSS-SR and IES. Journal of Traumatic Stress. 19 (1): 119-128.
5. Neal, L.A., Walter, B., Rollins, J., et al. (1994). Convergent Validity of Measures of Post-Traumatic Stress Disorder in a Mixed Military and Civilian Population. Journal of Traumatic Stress. 7 (3): 447-455.
6. Asukai, N. Kato, H. et al. (2002). Reliability and validity of the Japanese-language version of the Impact of event scale-revised (IES-R-J). Journal of Nervous and Mental Disease. 190 (3): 175-182.
7. Creamer, M. Bell, R. & Falilla, S. (2002). Psychometric properties of the Impact of Event Scale-Revised. Behaviour Research and Therapy. 41: 1489-1496.
8. Kawamura, N. Yoshiharu, K. & Nozomu, A. (2001) Suppression of Cellular Immunity in Men with a Past History of Post Traumatic Stress Disorder. American Journal of Psychiatry. 158: 484-486

Appendix 4. This appendix was part of the submitted manuscript and has been peer reviewed. It is posted as supplied by the authors

The Informant Questionnaire on Cognitive Decline in the Elderly (IQCODE; short version)[1]

This test is designed for use by professionals and patients able to speak English fluently.

Instructions

This assessment is directed at the patient's carer, family member or friend and is designed for them to complete. Generally, this test is completed without interference by a doctor or nurse, but it can be talked through with them if they need clarification.

Please give them page 2 and 3, and ask them to follow the instructions for completing the table.

Patient's name: ____

Date of birth: _____/_____/______

Hospital number: __________________

Name of person conducting assessment: _______________________________

Job title: ____

Date of assessment: _____/_____/______

The assessment

Now we want you to remember what your friend or relative was like 10 years ago and to compare it with what he/she is like now. 10 years ago was 19__. On the next page are situations where this person has to use his/her memory or intelligence and we want you to indicate whether this has improved, stayed the same or got worse than in that situation over the past 10 years. Note the importance of comparing his/her present performance with 10 years ago. So if 10 years ago this person always forgot where he/she had left things and he/she still does this, then this would be considered ‘Not much change’. Please indicate the changes you have observed by circling the appropriate answer.

This toolkit is supported by an educational grant from Shire Pharmaceuticals Ltd.
© Shire Pharmaceuticals Ltd, August 2006.
032/0711

		1	2	3	4	5
1	Remembering things about family and friends, eg occupations, birthdays, addresses	Much improved	A bit improved	Not much change	A bit worse	Much worse
2	Remembering things that have happened recently	Much improved	A bit improved	Not much change	A bit worse	Much worse
3	Recalling conversations a few days later	Much improved	A bit improved	Not much change	A bit worse	Much worse
4	Remembering her/his address and telephone number	Much improved	A bit improved	Not much change	A bit worse	Much worse
5	Remembering what day and month it is	Much improved	A bit improved	Not much change	A bit worse	Much worse
6	Remembering where things are usually kept	Much improved	A bit improved	Not much change	A bit worse	Much worse
7	Remembering where to find things which have been put in a different place from usual	Much improved	A bit improved	Not much change	A bit worse	Much worse
8	Knowing how to work familiar machines around the house	Much improved	A bit improved	Not much change	A bit worse	Much worse
9	Learning to use a new gadget or machine around the house	Much improved	A bit improved	Not much change	A bit worse	Much worse
10	Learning new things in general	Much improved	A bit improved	Not much change	A bit worse	Much worse
11	Following a story in a book or on TV	Much improved	A bit improved	Not much change	A bit worse	Much worse
12	Making decisions on everyday matters	Much improved	A bit improved	Not much change	A bit worse	Much worse
13	Handling money for shopping	Much improved	A bit improved	Not much change	A bit worse	Much worse
14	Handling financial matters, eg the pension, dealing with the bank	Much improved	A bit improved	Not much change	A bit worse	Much worse
15	Handling other everyday arithmetic problems, eg knowing how much food to buy, knowing how long between visits from family or friends	Much improved	A bit improved	Not much change	A bit worse	Much worse
16	Using his/her intelligence to understand what's going on and to reason things through	Much improved	A bit improved	Not much change	A bit worse	Much worse

Patient's name:_______________________________________ **Date of birth:** _____/_____/_____

This toolkit is supported by an educational grant from Shire Pharmaceuticals Ltd.
© Shire Pharmaceuticals Ltd, August 2006.
032/0711

[Do not leave this section with the patient's carer, family member or friend]

Patient's name: ______________________________

Date of birth: ____/____/____

Scoring the test

1 = Much improved
2 = A bit improved
3 = Not much change
4 = A bit worse
5 = Much worse

	Score for this question
1	
2	
3	
4	
5	
6	
7	
8	
9	
10	
11	
12	
13	
14	
15	
16	

Total score [ ]

To score the test, add up the result of each question (ie if 'Much improved' the result is 1 for that question), then divide this number by the total number of questions.

Record the final score of the test in the box below and also add this score to the patient's cognitive assessment record form.

$$\frac{\textbf{Sum of the results of all of the questions}}{\textbf{The total number of questions}} = \frac{\quad}{\textbf{16}} = \boxed{\quad}$$

Please add the score to the patient's cognitive assessment record form and go to table 3 in 'An introduction to the cognitive tests' for the cut-off point for a positive result. To determine the next steps required, please refer to the cognitive screening algorithm, which can be found in 'An introduction to the cognitive tests' or in the consensus statement.

Reference

1. Jorm AF. A short form of the Informant Questionnaire on Cognitive Decline in the Elderly (IQCODE): development and cross-validation. Psychol Med 1994; 24: 145–153.

Reproduced with the kind permission of Anthony Jorm.

This toolkit is supported by an educational grant from Shire Pharmaceuticals Ltd.
© Shire Pharmaceuticals Ltd, August 2006.
032/0711

Appendix 5. This appendix was part of the submitted manuscript and has been peer reviewed. It is posted as supplied by the authors

Regression analysis MMSE at discharge

Coefficients[a]

Model		Unstandardized Coefficients B	Unstandardized Coefficients Std. Error	Standardized Coefficients Beta	t	Sig.	95.0% Confidence Interval for B Lower Bound	95.0% Confidence Interval for B Upper Bound	Collinearity Statistics Tolerance	Collinearity Statistics VIF
1	(Constant)	3.312	.027		123.456	.000	3.259	3.365		
	CAMStatus	-.129	.045	-.277	-2.892	.005	-.218	-.041	1.000	1.000
2	(Constant)	3.550	.086		41.448	.000	3.380	3.720		
	CAMStatus	-.127	.044	-.271	-2.911	.004	-.213	-.040	.988	1.012
	Age	-.004	.001	-.244	-2.620	.010	-.006	-.001	.986	1.014
	Gender	-.050	.042	-.111	-1.186	.239	-.133	.033	.983	1.017

a. Dependent Variable: LNMMSE

Bootstrap for Coefficients

Model		B	Bootstrap[a] Bias	Bootstrap[a] Std. Error	Bootstrap[a] Sig. (2-tailed)	Bootstrap[a] BCa 95% Confidence Interval Lower	Bootstrap[a] BCa 95% Confidence Interval Upper
1	(Constant)	3.312	.000	.014	.001	3.278	3.341
	CAMStatus	-.129	-.003	.057	.041	-.263	-.026
2	(Constant)	3.550	.002	.078	.001	3.429	3.720
	CAMStatus	-.127	-.003	.054	.030	-.244	-.033
	Age	-.004	-1.534E-5	.001	.014	-.007	-.001
	Gender	-.050	-.001	.044	.258	-.134	.037

a. Unless otherwise noted, bootstrap results are based on 1000 bootstrap samples

Appendix 6. This appendix was part of the submitted manuscript and has been peer reviewed. It is posted as supplied by the authors

Regression analysis IES 12 months (Ln-transformed)

Coefficients[a]

Model		Unstandardized Coefficients		Standardized Coefficients	t	Sig.	95.0% Confidence Interval for B		Collinearity Statistics	
		B	Std. Error	Beta			Lower Bound	Upper Bound	Tolerance	VIF
1	(Constant)	2.023	.438		4.616	.000	1.142	2.904		
	CAMStatus	.593	.301	.274	1.969	.055	-.012	1.199	.935	1.070
	Apache	.026	.021	.177	1.269	.211	-.015	.068	.935	1.070

a. Dependent Variable: LNIES12

Bootstrap for Coefficients

Model		B	Bootstrap[a]				
			Bias	Std. Error	Sig. (2-tailed)	BCa 95% Confidence Interval	
						Lower	Upper
1	(Constant)	2.023	.003	.459	.001	1.134	2.960
	CAMStatus	.593	.004	.300	.056	.018	1.169
	Apache	.026	-.001	.020	.159	-.016	.062

a. Unless otherwise noted, bootstrap results are based on 1000 bootstrap samples
